# Supplementary material for: Impact of transport electrification on critical metal sustainability with a focus on the heavy-duty segment
Source: Nat Commun. 2019 Nov 27;10:5398. doi: 10.1038/s41467-019-13400-1 (PMC6881386; doi:10.1038/s41467-019-13400-1)
Supplement: Supplementary file 2 — Supplementary Information [file 41467_2019_13400_MOESM2_ESM.pdf]

Supplementary Information for

Impact of transport electrification on critical metal sustainability with a focus on the heavy-duty segment

Hao *et al.*

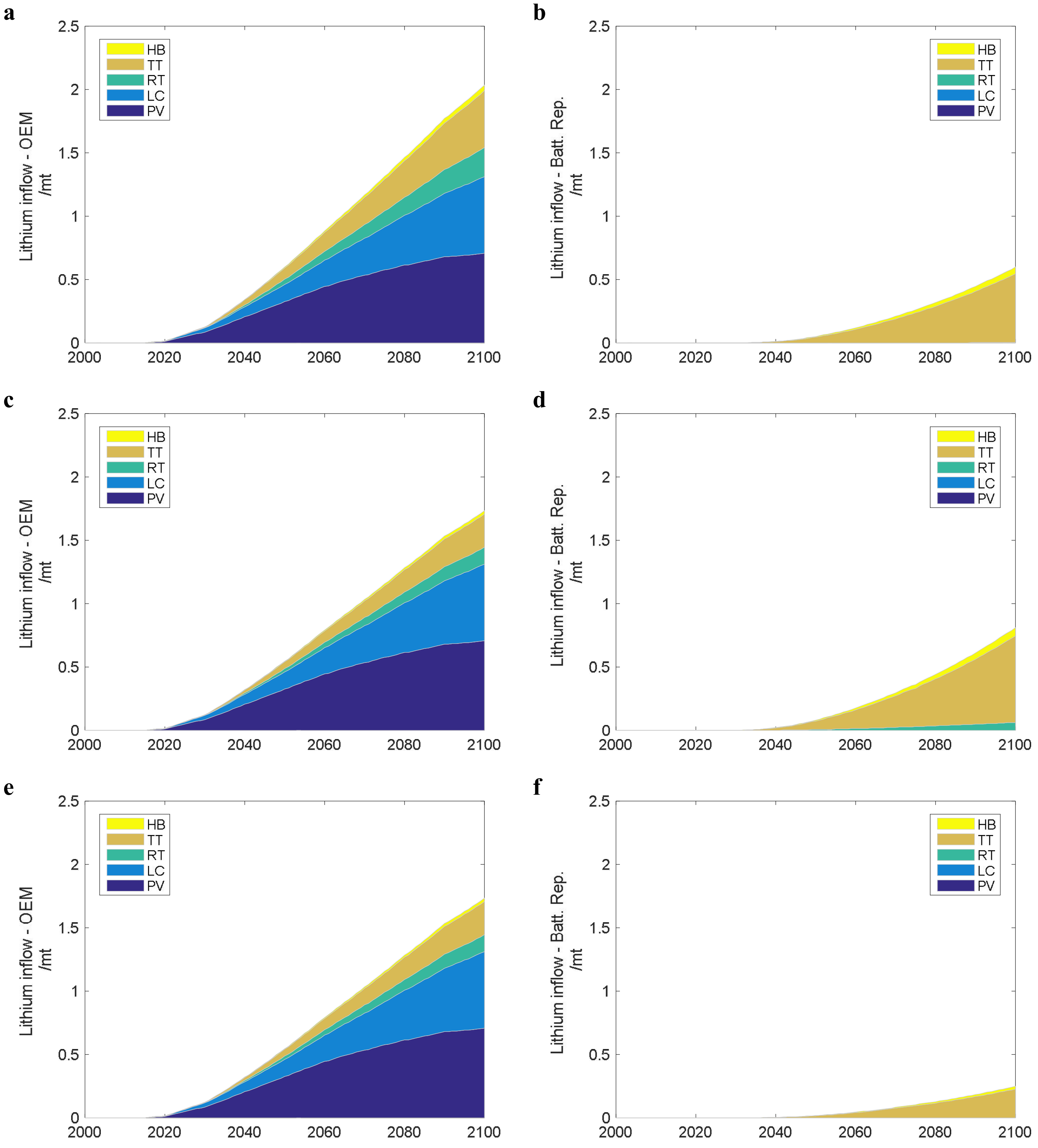

Supplementary Figure 1 Lithium inflow to global vehicle fleet. The subfigures indicate lithium inflow associated with vehicle manufacturing under scenario D2 (**a**), D3 (**c**), D4 (**e**); lithium inflow associated with battery replacement under scenario D2 (**b**), D3 (**d**), D4 (**f**). For ease of comparison, all figures are plotted using the same vertical axis scales. PV: Passenger Vehicle; LC: Light-duty Commercial vehicle; RT: Medium-duty truck; TT: Heavy-duty truck; HB: Heavy-duty Bus. Source data are provided as a Source Data file.

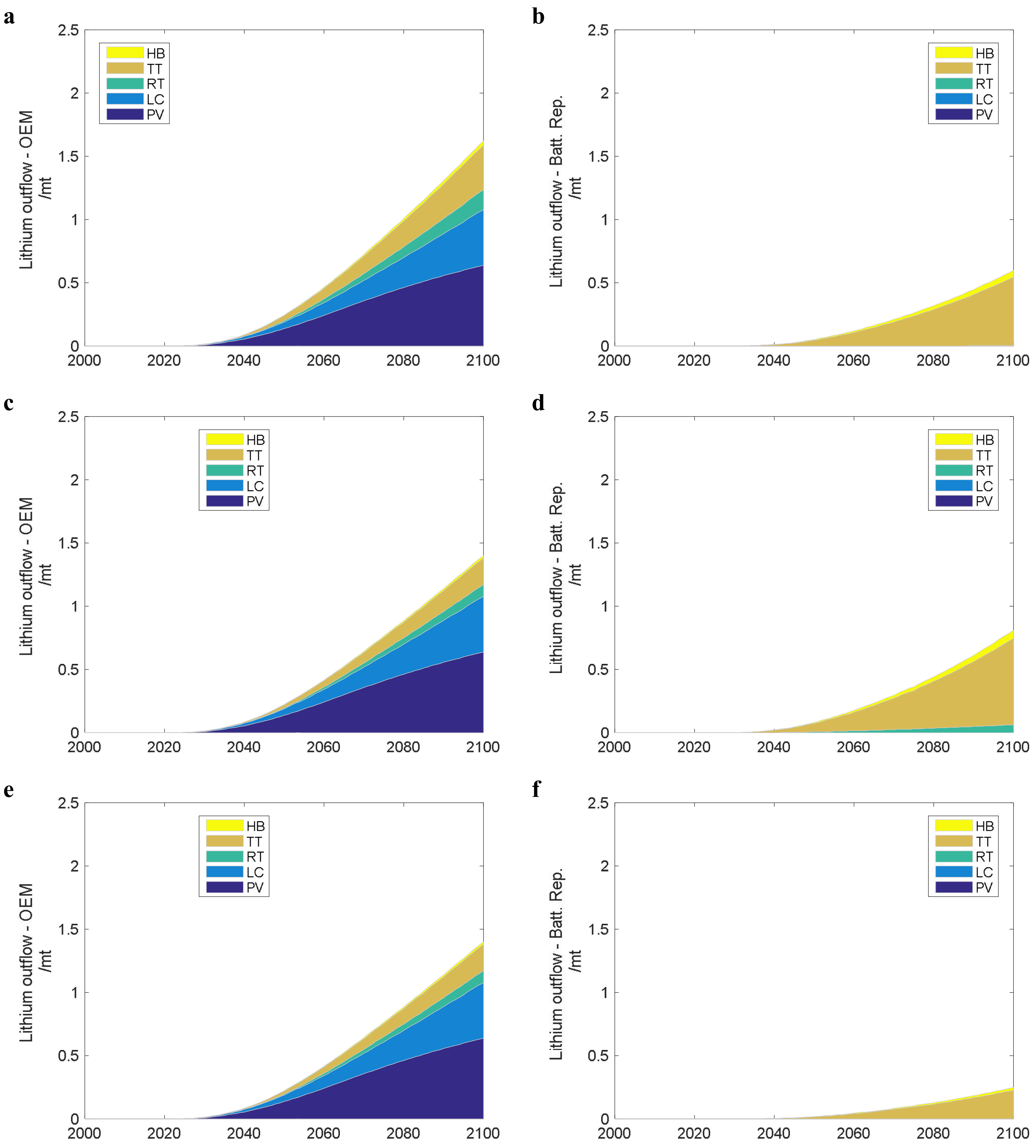

Supplementary Figure 2 Lithium outflow from global vehicle fleet. The subfigures indicate lithium outflow associated with end-of-life vehicles under scenario D2 (**a**), D3 (**c**), D4 (**e**); lithium outflow associated with battery replacement under scenario D2 (**b**), D3 (**d**), D4 (**f**). For ease of comparison, all figures are plotted using the same vertical axis scales. Source data are provided as a Source Data file.

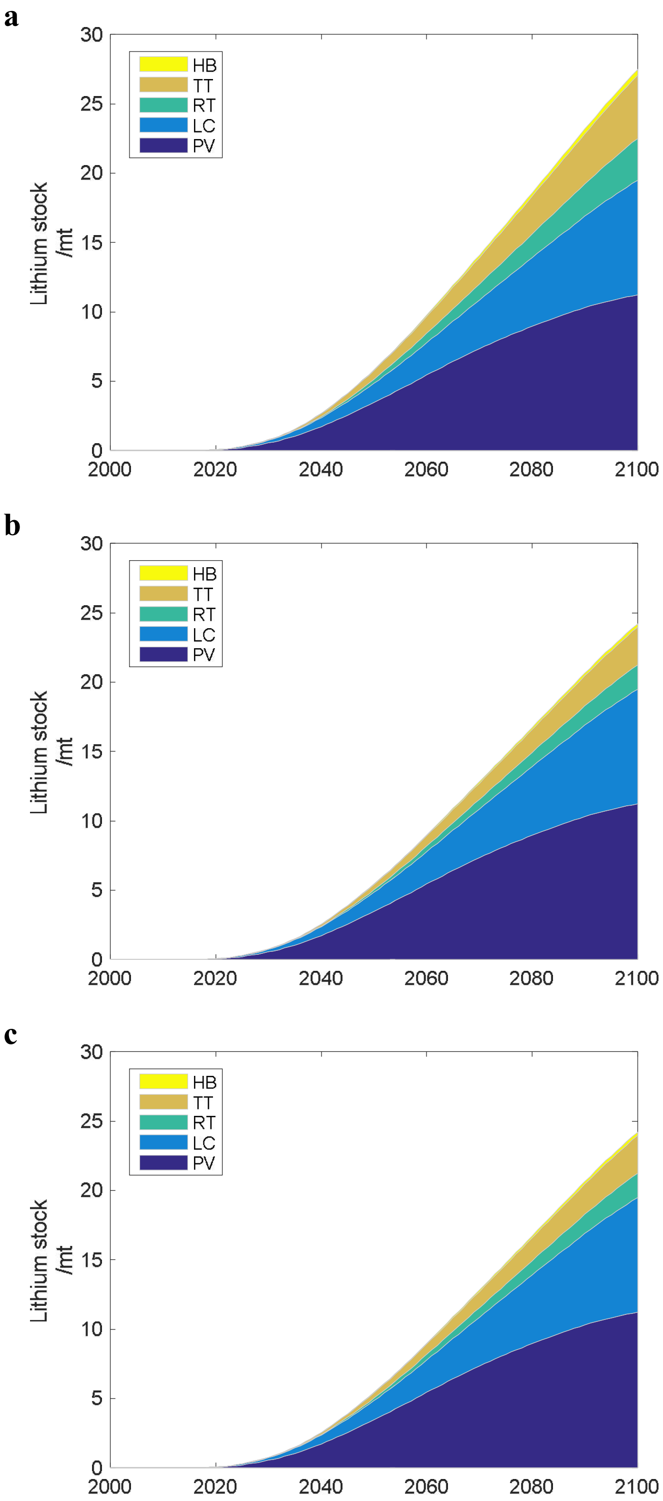

Supplementary Figure 3 Lithium stock in global vehicle fleet. The subfigures indicate lithium stock under scenario D2 (**a**), D3 (**b**), D4 (**c**). Source data are provided as a Source Data file.

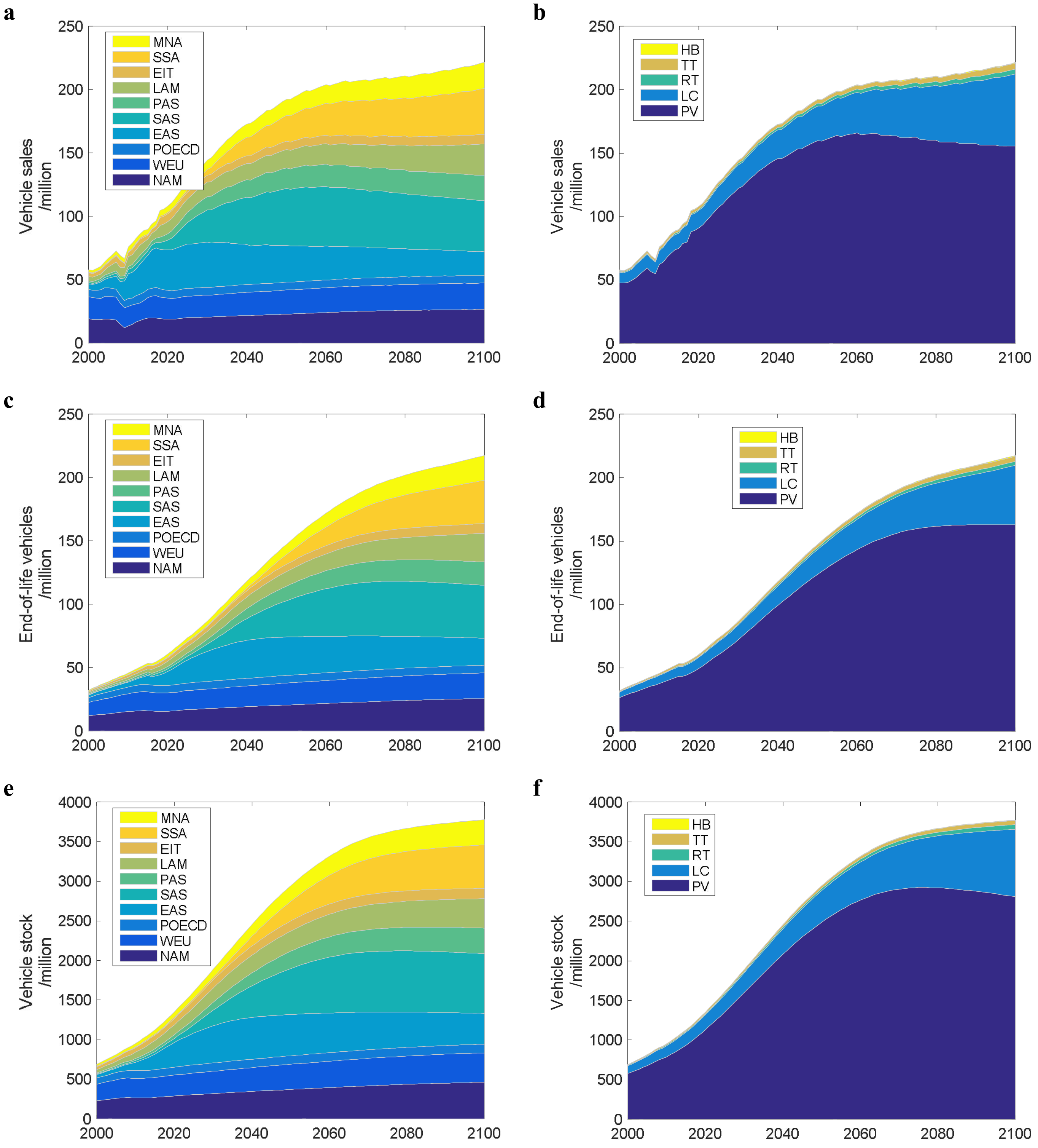

Supplementary Figure 4 Assumptions for global vehicle sales by region (**a**)/vehicle type (**b**), scrappage by region (**c**)/vehicle type (**d**) and stock by region (**e**)/vehicle type (**f**). The region categorization is based on IPCC RC10 [1]. NAM: North America; WEU: Western Europe; POECD: Pacific OECD; EAS: East Asia; SAS: South Asia; PAS: South-East Asia and Pacific; LAM: Latin America and Caribbean; EIT: Economies in Transition; SSA: Sub-Saharan Africa; MNA: Middle East and North Africa. Source data are provided as a Source Data file.

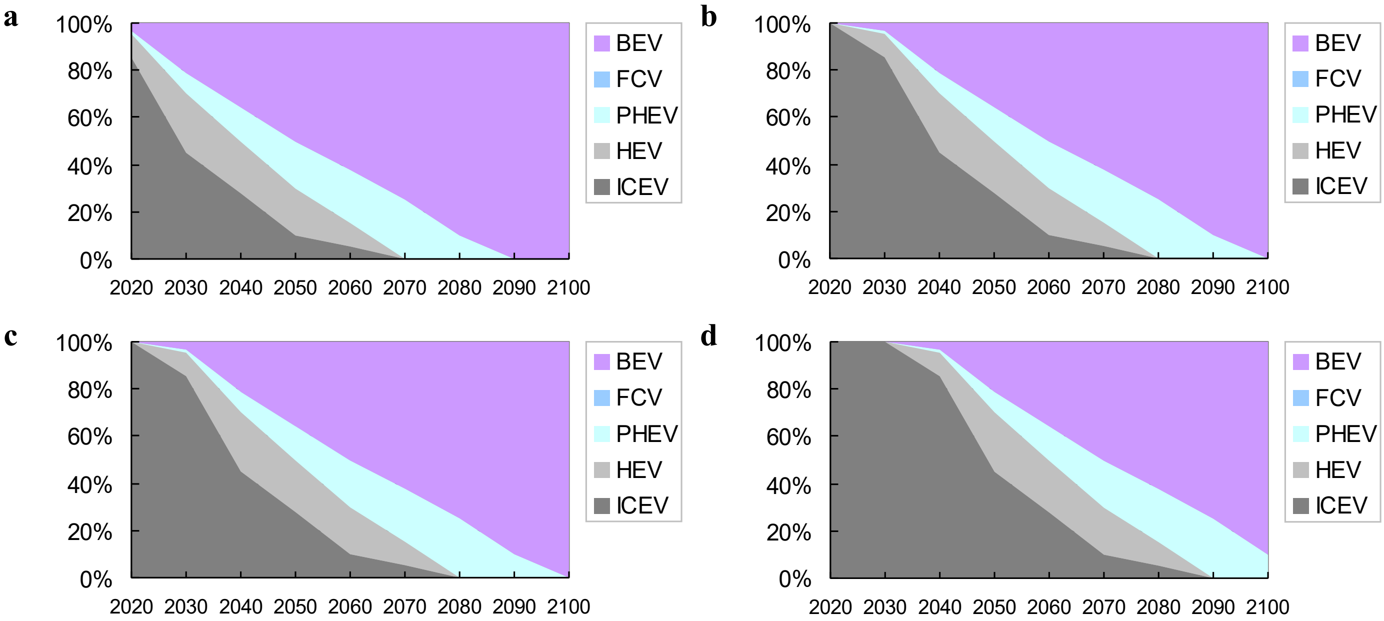

Supplementary Figure 5 Assumptions for market penetration of advanced vehicle powertrain technologies. The subfigures indicate powertrain mix of LDVs in MDCs (**a**), LDVs in LDCs (**b**), HDVs in MDCs (**c**) and HDVs in LDCs (**d**). China, as an exception, uses the MDC assumptions considering its ambitious target in promoting PEVs. LDV: Light-duty vehicle (PV+LC); HDV: Heavy-duty vehicle (RT+TT+HB); MDC: More Developed Countries (NAM+WEU+POECD); LDC: Less Developed Countries (EAS+SAS+PAS+LAM+EIT+SSA+MNA); ICEV: Internal Combustion Engine Vehicle; HEV: Hybrid Electric Vehicle; PHEV: Plug-in Hybrid Electric Vehicle; FCV: Fuel Cell Vehicle; BEV: Battery Electric Vehicle. Source data are provided as a Source Data file.

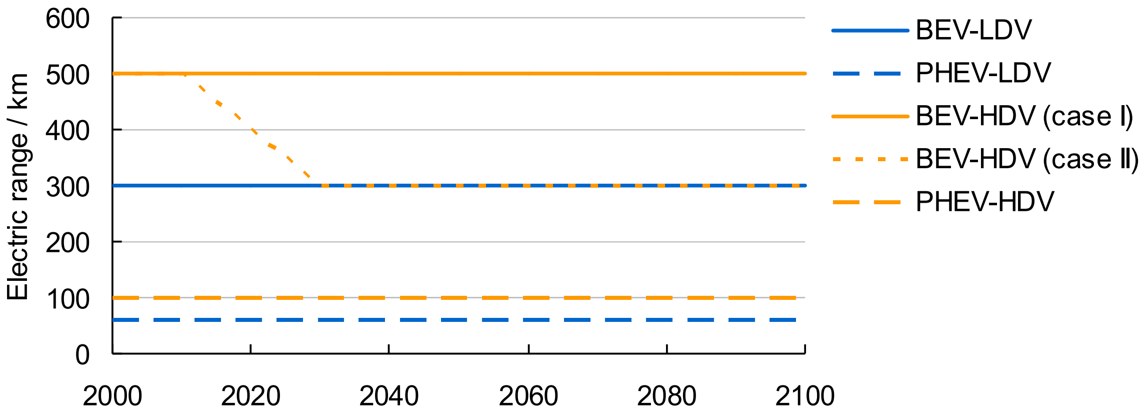

Supplementary Figure 6 The assumptions for vehicle electric range. Source data are provided as a Source Data file.

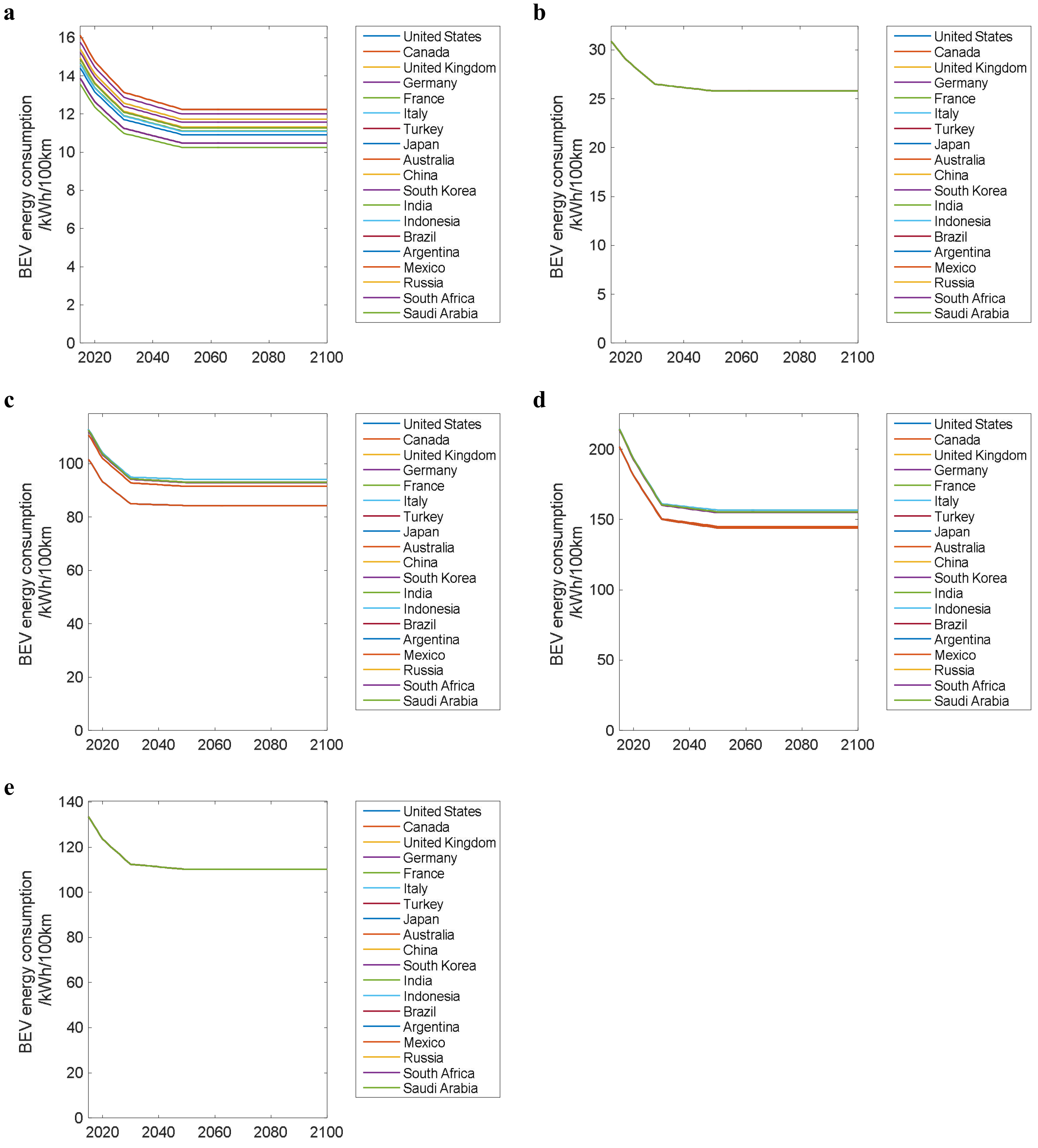

Supplementary Figure 7 The energy consumption rates of BEVs. The subfigures indicate energy consumption rates for PV (**a**), LC (**b**), RT (**c**), TT (**d**), and HB (**e**). The results are based on the case I electric range assumptions. Data are collected with the authors' best efforts from a wide range of literatures and industry experts [2, 3, 4, 5, 6, 7]. A geographic similarity approach is taken, that is, the vehicle specifications in countries other than G20 countries follow the G20 country in the same geographic region. There is little country-specific vehicle specifications for LC and HB, for which their vehicle specifications are assumed to be the same globally. Source data are provided as a Source Data file.

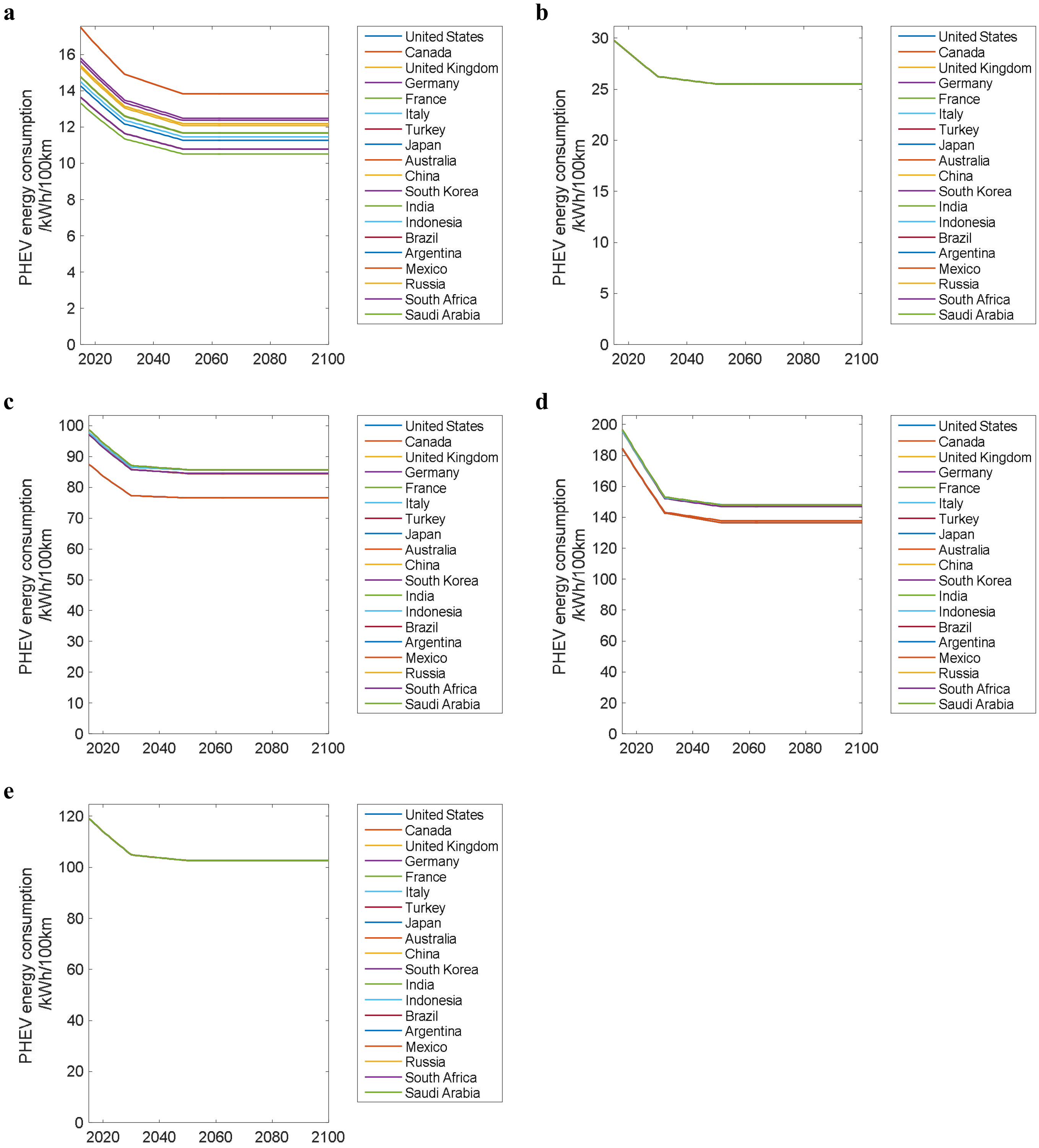

Supplementary Figure 8 The energy consumption rates of PHEVs. The subfigures indicate energy consumption rates for PV (**a**), LC (**b**), RT (**c**), TT (**d**), and HB (**e**). Source data are provided as a Source Data file.

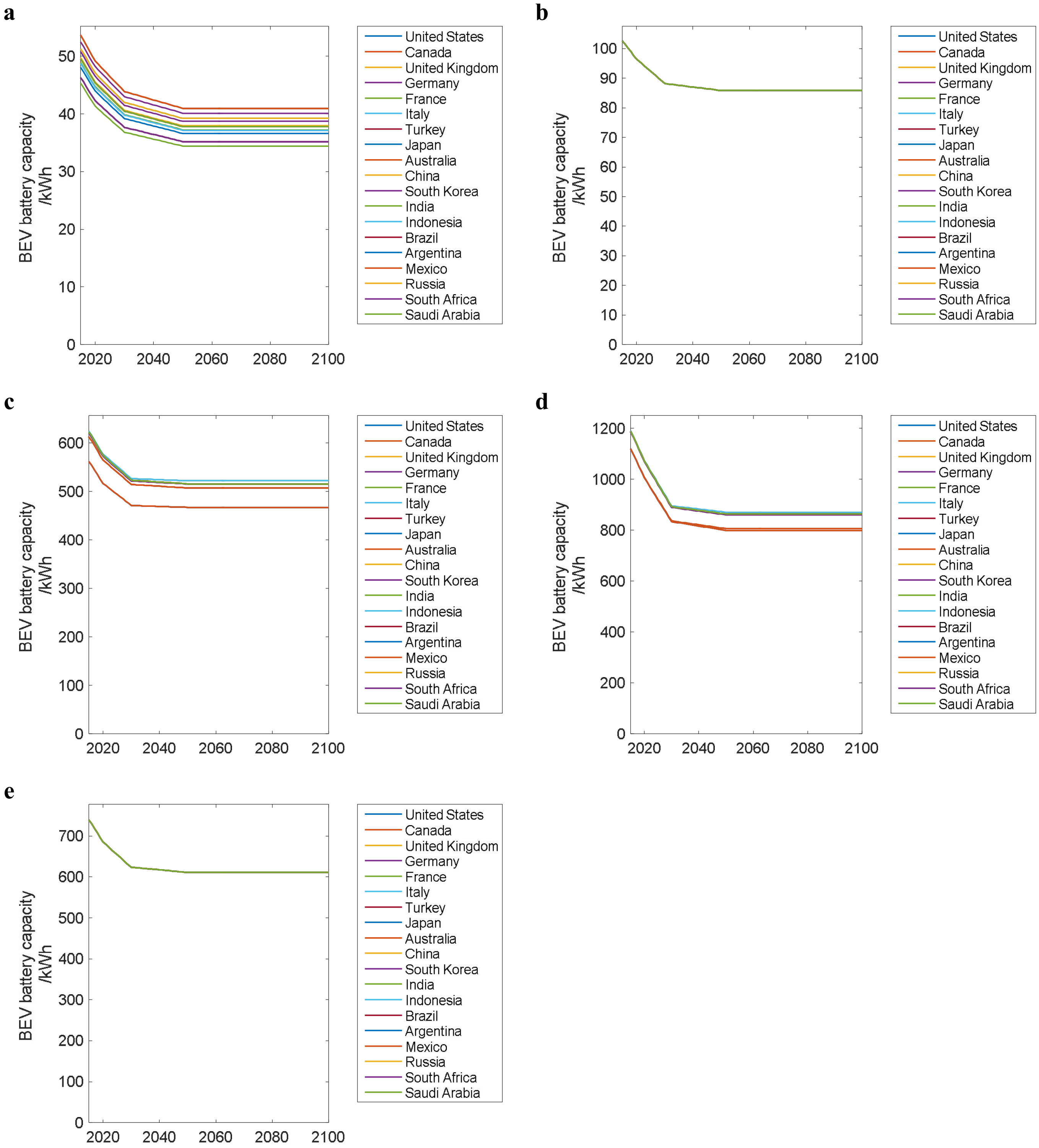

Supplementary Figure 9 The battery capacities of BEVs. The subfigures indicate battery capacities for PV (**a**), LC (**b**), RT (**c**), TT (**d**), and HB (**e**). The results are based on the case I electric range assumptions. Source data are provided as a Source Data file.

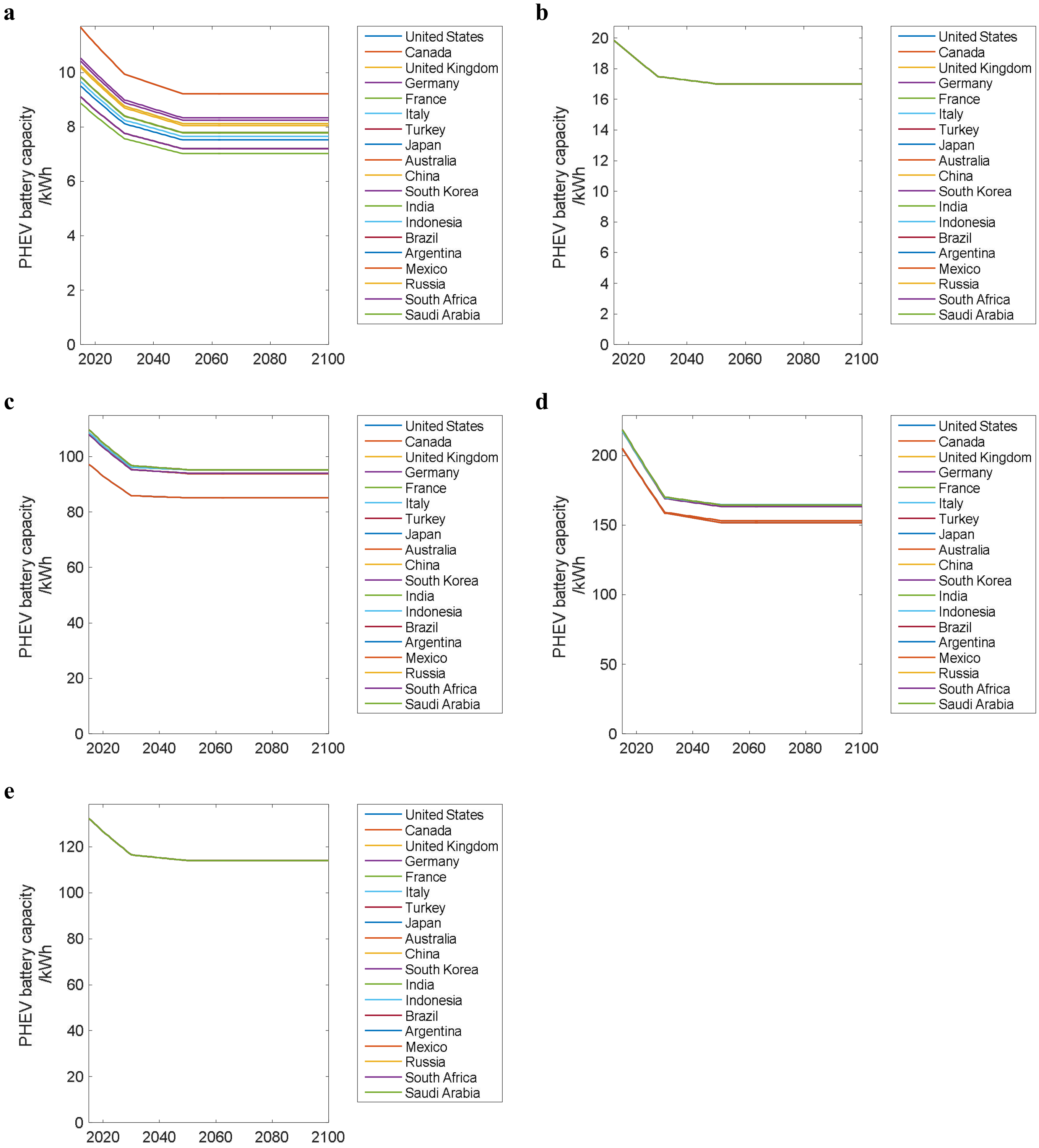

Supplementary Figure 10 The battery capacities of PHEVs. The subfigures indicate battery capacities for PV (**a**), LC (**b**), RT (**c**), TT (**d**), and HB (**e**). Source data are provided as a Source Data file.

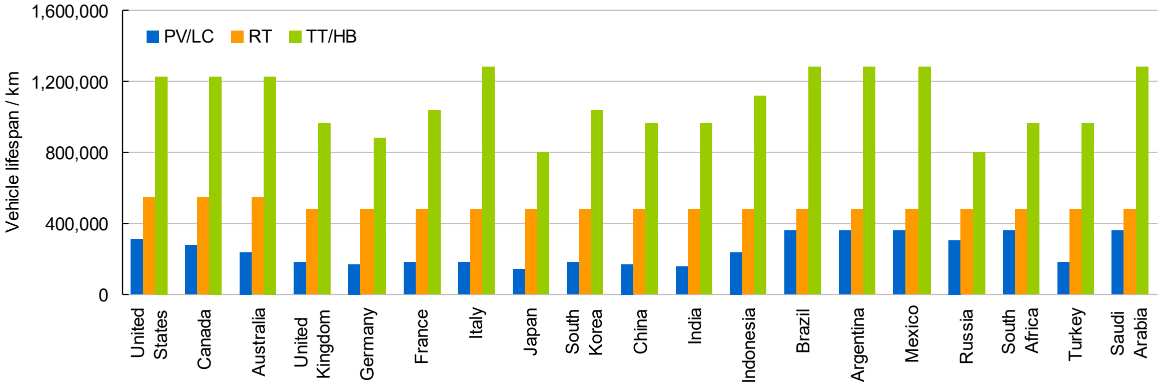

Supplementary Figure 11 The vehicle lifespan assumptions. Data are collected with the authors' best efforts [6, 8, 9, 10]. Due to lack of data, a geographic similarity approach is taken to estimate vehicle lifespan in countries where data is not available. To ensure data quality, the investigated country-specific vehicle lifespans are further coupled with the country-specific vehicle energy consumption rate data to calculate the country-specific energy demand from the road transport sector. These estimated energy demands are compared with existing energy statistics to further calibrate the vehicle lifespans. Source data are provided as a Source Data file.

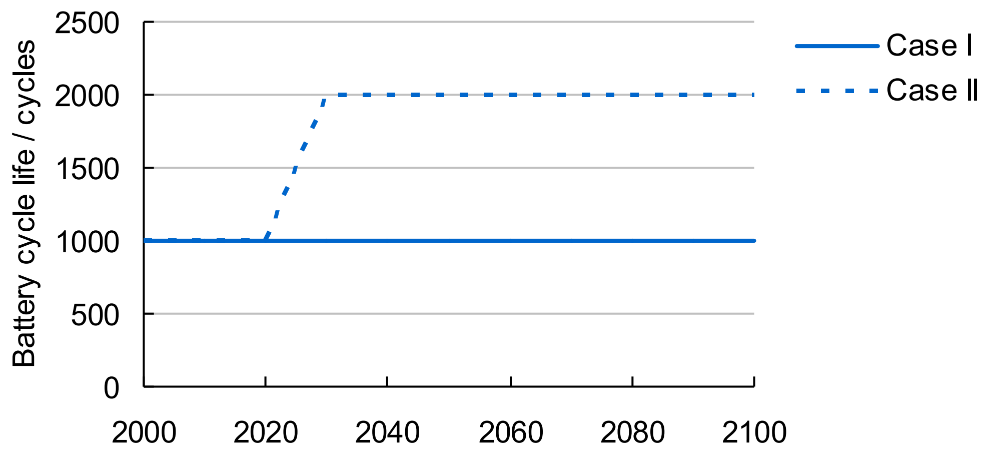

Supplementary Figure 12 The assumptions for battery cycle life. Source data are provided as a Source Data file.

Supplementary Table 1 A summarization of the literatures investigating the resource constraints for vehicle electrification and their major conclusions.

Author	Year	Resource implications		
		Lithium	Cobalt	Nickel
Ziemann et al.	2018	Lithium recovery from EV battery recycling could result in a significant oversupply of secondary material if its quality is not high enough to allow for reprocessing in battery production.		
Deetman et al.	2018	The demand for lithium and cobalt is expected to increase by a factor 10 to more than 20, as a result of future (hybrid) electric car purchases.		
Vaalma et al.	2018	A comparison of the calculated demands with today's identified reserves does not indicate that a depletion of the identified reserves will occur in the short to medium term.	Today's identified reserves are already found to be strained with regard to the accumulated production of 35 years.	For nickel, it has already been reported that there is a supply risk in the medium to long term owing to the demand of other industries.
Helbig et al.	2018	In our set of ten elements, a substantial future demand was only identified for lithium and cobalt with growths of 390% and 90%.		An increase in use of the eight other elements for rapidly evolving future technologies could occur as well, but not on a large scale.
Harvey	2018	Cumulative Li demand by 2100 in scenarios that see a complete transition of the global LDV fleet to EVs could exceed the usable Li resource.	There have been almost no assessments of supply constraints on Co, which is used in NCA and NCM batteries.	
de Koning et al.	2018	For lithium, the current economic reserves as known in 2000 are not		The high estimate of cumulative demand for nickel until 2050 can be

		sufficient to meet the cumulative demand for these metals until 2050.		met by the currently known economic reserves of metals.
Pehlken et al.	2017	With a view to currently known reserves, the cumulative demand for battery technology projected in the dominant scenario will consume 74–248 % (for two different cases) of the lithium reserves by 2050.	With a view to currently known reserves, the cumulative demand for battery technology projected in the dominant scenario will consume 50 % of the cobalt reserves by 2050.	
Olivetti et al.	2017	The availability of Li has proved to be a controversial topic.	Our analysis finds that while Co supply will meet demand for the lower estimates of demand for LIBs, there is a potential for availability concern if there is rapid vehicle adoption.	Even the high demand is only 22% of Ni production in 2015.
Narins	2017	Ultimately, the rise of the global electric car industry will not be constrained by lithium availability.		
Martin et al.	2017	These projects with an annual production volume ≥20,000 t LCE will be able to extract 169,000 t LCE per year and consequently should meet the requested demand.		
Sverdrup	2016	If our basic simulation assumptions are right, the lithium resources will be largely exhausted by 2400.		
Simon et al.	2015	In the case of a hypothetical European production of future traction battery	Demand on cobalt and manganese are found to be far below the available	In the case of a hypothetical European production of future

		cells, shortage in European lithium reserves might be expectable at around 2025.	European reserves.	traction battery cells, shortage in European nickel reserves might be expectable at around 2025.
Speirs et al.	2014	Under this rate of production growth, it is plausible that lithium supply will meet increasing lithium demand over the coming decades to 2050.		
Richa et al.	2014	A number of uncertainties still exist, and exact estimation of future waste flows will depend on the ability to further refine the forecasts of EV sales, battery and EV lifespan, and trajectories of battery technology deployment.		
Delucchi et al.	2014	If very large numbers of EVs are manufactured for many decades, then in the long term some of the rarer materials, such as neodymium, platinum and lithium (in batteries), will have to be recycled or eventually replaced with less-scarce materials unless additional resources are located.	The demand for cobalt for the production of 20 million batteries per year would be about equal to current world mine production of cobalt, and would deplete current cobalt reserves in less than 60 years (and deplete cobalt resources in about 120 years)	The demand for nickel to make 20 million EV batteries per year would be two orders of magnitude larger than current world mine production.
Vikström et al.	2013	We find that the availability of lithium could in fact be a problem for fulfilling this scenario if lithium-ion batteries are to be used.		
Miedema et al.	2013	The results of this research show that undersupply can be expected in the EU27 until 2045 somewhere between 0.5 Mt and 2.8 Mt.		
Mohr et	2012	The lithium market can expand for		

al.		several decades with no shortages in lithium likely.		
Kushnir et al.	2012	The presently known lithium resources excluding the ocean will only be exhausted this century if large scale use of predominantly BEV sized batteries comes into play, or if batteries are not recycled. This same resource will extend into next century for low vehicle count scenarios with plug in hybrids and high recycling rates.		
Keoleian et al.	2012	Although demand for lithium could increase significantly, its supply is ample to meet demand well beyond midcentury.	Cobalt, used in nickel-metal hydride (NiMH) batteries for current hybrid electric vehicles, is not expected to reach a critical supply level in the next 15 years.	
Grosjean et al.	2012	This is a very reassuring figure that comforts us in the idea that there is globally enough lithium on Earth to supply.		
Wanger	2011	I use cumulative data of vehicle, mobile phone, laptop, and digital camera production to show that demand will overshoot the available global Lithium resources before 2025.		
Gruber et	2011	We conclude that even with a rapid and		

al.		widespread adoption of electric vehicles powered by lithium-ion batteries, lithium resources are sufficient to support demand until at least the end of this century.		
Yaksic et al.	2009	The shape of the lithium cumulative availability curve indicates that depletion is not likely to pose a serious problem over the rest of this century and well beyond.		
Gaines et al.	2009	It is reasonable to expect the lithium production industry to be able to expand at the relatively slow rate required to meet automotive battery demand.	If NCA-G were the only chemistry used, cobalt use could make a dent in the reserve base by 2050.	

Note: the table is summarized based on references [11, 12, 13, 14, 15, 16, 17, 18, 19, 20, 21, 22, 23, 24, 25, 26, 27, 28, 29, 30, 31, 32, 33, 34, 35].

Supplementary Table 2 The accumulated lithium demand and recycling under different scenarios.

		**Vehicle manuf.**				**Battery replacement**				**Total (Vehicle manuf. + battery replacement)**			
		D1	D2	D3	D4	D1	D2	D3	D4	D1	D2	D3	D4
**Inflow (Gross demand)**	PV	32.9	32.9	32.9	32.9	0.0	0.0	0.0	0.0	32.9	32.9	32.9	32.9
	LC	19.7	19.7	19.7	19.7	0.0	0.0	0.0	0.0	19.7	19.7	19.7	19.7
	RT	0.0	6.9	4.1	4.1	0.0	0.1	1.6	0.0	0.0	7.0	5.7	4.1
	TT	0.0	13.9	8.4	8.4	0.0	13.3	17.3	5.4	0.0	27.3	25.7	13.9
	HB	0.0	1.3	0.8	0.8	0.0	1.4	1.7	0.5	0.0	2.7	2.5	1.3
	**LDV**	**52.6**	**52.6**	**52.6**	**52.6**	**0.0**	**0.0**	**0.0**	**0.0**	**52.6**	**52.6**	**52.6**	**52.6**
	**HDV**	**0.0**	**22.2**	**13.3**	**13.3**	**0.0**	**14.8**	**20.6**	**6.0**	**0.0**	**36.9**	**33.9**	**19.3**
	**Total**	**52.6**	**74.7**	**65.8**	**65.8**	**0.0**	**14.8**	**20.7**	**6.0**	**52.6**	**89.5**	**86.5**	**71.8**
**Outflow**	PV	21.7	21.7	21.7	21.7	0.0	0.0	0.0	0.0	21.7	21.7	21.7	21.7
	LC	11.4	11.4	11.4	11.4	0.0	0.0	0.0	0.0	11.4	11.4	11.4	11.4
	RT	0.0	3.8	2.3	2.3	0.0	0.1	1.6	0.0	0.0	3.9	3.9	2.3
	TT	0.0	9.4	5.7	5.7	0.0	13.3	17.3	5.4	0.0	22.7	23.0	11.2
	HB	0.0	0.9	0.5	0.5	0.0	1.4	1.7	0.5	0.0	2.3	2.2	1.1
	**LDV**	**33.1**	**33.1**	**33.1**	**33.1**	**0.0**	**0.0**	**0.0**	**0.0**	**33.1**	**33.1**	**33.1**	**33.1**
	**HDV**	**0.0**	**14.1**	**8.5**	**8.5**	**0.0**	**14.8**	**20.6**	**6.0**	**0.0**	**28.9**	**29.2**	**14.5**
	**Total**	**33.1**	**47.2**	**41.6**	**41.6**	**0.0**	**14.8**	**20.7**	**6.0**	**33.1**	**62.0**	**62.3**	**47.6**
**Recycling**	PV	17.3	17.3	17.3	17.3	0.0	0.0	0.0	0.0	17.4	17.4	17.4	17.3
	LC	9.1	9.1	9.1	9.1	0.0	0.0	0.0	0.0	9.1	9.1	9.1	9.1
	RT	0.0	3.1	1.8	1.8	0.0	0.1	1.3	0.0	0.0	3.1	3.1	1.8
	TT	0.0	7.5	4.6	4.6	0.0	10.7	13.8	4.4	0.0	18.2	18.4	8.9
	HB	0.0	0.7	0.4	0.4	0.0	1.1	1.4	0.4	0.0	1.8	1.8	0.9

	**LDV**	**26.5**	**26.5**	**26.5**	**26.5**	**0.0**	**0.0**	**0.0**	**0.0**	**26.5**	**26.5**	**26.5**	**26.5**
	**HDV**	**0.0**	**11.3**	**6.8**	**6.8**	**0.0**	**11.8**	**16.5**	**4.8**	**0.0**	**23.1**	**23.3**	**11.6**
	**Total**	**26.5**	**37.8**	**33.3**	**33.3**	**0.0**	**11.8**	**16.5**	**4.8**	**26.5**	**49.6**	**49.8**	**38.1**
**Net demand**	PV	15.6	15.6	15.6	15.6	0.0	0.0	0.0	0.0	15.6	15.6	15.6	15.6
	LC	10.5	10.5	10.5	10.5	0.0	0.0	0.0	0.0	10.5	10.5	10.5	10.5
	RT	0.0	3.8	2.2	2.2	0.0	0.0	0.3	0.0	0.0	3.8	2.6	2.2
	TT	0.0	6.4	3.9	3.9	0.0	2.7	3.5	1.1	0.0	9.1	7.3	4.9
	HB	0.0	0.6	0.4	0.4	0.0	0.3	0.3	0.1	0.0	0.9	0.7	0.5
	**LDV**	**26.1**	**26.1**	**26.1**	**26.1**	**0.0**	**0.0**	**0.0**	**0.0**	**26.1**	**26.1**	**26.1**	**26.1**
	**HDV**	**0.0**	**10.9**	**6.4**	**6.4**	**0.0**	**3.0**	**4.1**	**1.2**	**0.0**	**13.8**	**10.6**	**7.6**
	**Total**	**26.1**	**36.9**	**32.5**	**32.5**	**0.0**	**3.0**	**4.1**	**1.2**	**26.1**	**39.9**	**36.7**	**33.7**

Supplementary Table 3 The basic assumptions behind the demand scenarios.

		**Scenario D1**	**Scenario D2**	**Scenario D3**	**Scenario D4**
Market penetration of PEVs	Case I (LDV)	√			
	Case II (LDV+HDV)		√	√	√
Electric range	Case I (Normal electric range)	√	√		
	Case II (Reduced electric range)			√	√
Battery durability	Case I (Unchanged battery durability)	√	√	√	
	Case II (Improved battery durability)				√

Note: Four demand scenarios (D1/D2/D3/D4) are established to reflect future lithium demand under different market and technological circumstances.

Supplementary References

1. IPCC. Climate Change 2014: Mitigation of Climate Change. Intergovernmental Panel on Climate Change (2015). https://www.ipcc.ch/report/ar5/wg3/
2. ICCT. Market analysis of heavy-duty vehicles in India. International Council on Clean Transportation (2015). https://www.theicct.org/publications/market-analysis-heavy-duty-vehicles-india
3. ICCT. Brazil passenger vehicle market statistics. International Council on Clean Transportation (2015). https://www.theicct.org/publications/brazil-passenger-vehicle-market-statistics
4. ICCT. The automotive sector in Turkey: A baseline analysis. International Council on Clean Transportation (2016). https://www.theicct.org/publications/automotive-sector-turkey-baseline-analysis
5. ICCT. European vehicle market statistics, 2018/2019. International Council on Clean Transportation (2018). https://www.theicct.org/publications/european-vehicle-market-statistics-20182019
6. ORNL. Transportation Energy Data Book. Oak Ridge National Laboratory (2018). https://cta.ornl.gov/data/index.shtml
7. Hao, H., Geng, Y. & Sarkis, J. Carbon footprint of global passenger cars: Scenarios through 2050. *Energy* **101**, 121-131 (2016).
8. ICCT. Transitioning to zero-emission heavy-duty freight vehicles. International Council on Clean Transportation (2017). https://www.theicct.org/publications/transitioning-zero-emission-heavy-duty-freight-vehicles
9. ICCT. European Heavy-Duty Vehicles: Cost-Effectiveness of Fuel-Efficiency Technologies for Long-Haul Tractor-Trailers in the 2025-2030 Timeframe. International Council on Clean Transportation (2018). https://theicct.org/publications/cost-effectiveness-of-fuel-efficiency-tech-tractor-trailers
10. ATRI. An Analysis of the Operational Costs of Trucking: 2018 Update. American Transportation Research Institute (2018). https://truckingresearch.org/atri-research/operational-costs-of-trucking/
11. Gaines, L. & Nelson, P. Lithium-ion Batteries: Possible Materials Issues. Argonne National Laboratory (2009).
12. Yaksic, A. & Tilton, J. E. Using the cumulative availability curve to assess the threat of mineral depletion: The case of lithium. *Resources Policy* **34**, 185-194 (2009).
13. Gruber, P. W., Medina, P. A., Keoleian, G. A., Kesler, S. E., Everson, M. P. & Wallington, T. J. Global Lithium Availability. *Journal of Industrial Ecology* **15**, 760-775 (2011).
14. Wanger, T. C. The Lithium future—resources, recycling, and the environment. *Conservation Letters* **4**, 202-206 (2011).
15. Grosjean, C., Miranda, P. H., Perrin, M. & Poggi, P. Assessment of world lithium resources and consequences of their geographic distribution on the expected development of the electric vehicle industry. *Renewable and Sustainable Energy Reviews* **16**, 1735-1744 (2012).
16. Keoleian, G. A. & Sullivan, J. L. Materials challenges and opportunities for enhancing the sustainability of automobiles. *MRS Bulletin* **37**, 365-373 (2012).
17. Kushnir, D. & Sandén, B. A. The time dimension and lithium resource constraints for electric vehicles. *Resources Policy* **37**, 93-103 (2012).

18. Mohr, S. H., Mudd, G. M. & Giurco, D. Lithium Resources and Production: Critical Assessment and Global Projections. *Minerals* **2**, (2012).
19. Miedema, J. H. & Moll, H. C. Lithium availability in the EU27 for battery-driven vehicles: The impact of recycling and substitution on the confrontation between supply and demand until2050. *Resources Policy* **38**, 204-211 (2013).
20. Vikström, H., Davidsson, S. & Höök, M. Lithium availability and future production outlooks. *Applied Energy* **110**, 252-266 (2013).
21. Delucchi, M. A., Yang, C., Burke, A. F., Ogden, J. M., Kurani, K., Kessler, J. & Sperling, D. An assessment of electric vehicles: technology, infrastructure requirements, greenhouse-gas emissions, petroleum use, material use, lifetime cost, consumer acceptance and policy initiatives. *Philosophical Transactions of the Royal Society A: Mathematical, Physical and Engineering Sciences* **372**, 20120325 (2014).
22. Richa, K., Babbitt, C. W., Gaustad, G. & Wang, X. A future perspective on lithium-ion battery waste flows from electric vehicles. *Resources, Conservation and Recycling* **83**, 63-76 (2014).
23. Speirs, J., Contestabile, M., Houari, Y. & Gross, R. The future of lithium availability for electric vehicle batteries. *Renewable and Sustainable Energy Reviews* **35**, 183-193 (2014).
24. Simon, B., Ziemann, S. & Weil, M. Potential metal requirement of active materials in lithium-ion battery cells of electric vehicles and its impact on reserves: Focus on Europe. *Resources, Conservation and Recycling* **104**, 300-310 (2015).
25. Sverdrup, H. U. Modelling global extraction, supply, price and depletion of the extractable geological resources with the LITHIUM model. *Resources, Conservation and Recycling* **114**, 112-129 (2016).
26. Martin, G., Rentsch, L., Höck, M. & Bertau, M. Lithium market research – global supply, future demand and price development. *Energy Storage Materials* **6**, 171-179 (2017).
27. Narins, T. P. The battery business: Lithium availability and the growth of the global electric car industry. *The Extractive Industries and Society* **4**, 321-328 (2017).
28. Olivetti, E. A., Ceder, G., Gaustad, G. G. & Fu, X. Lithium-Ion Battery Supply Chain Considerations: Analysis of Potential Bottlenecks in Critical Metals. *Joule* **1**, 229-243 (2017).
29. Pehlken, A., Albach, S. & Vogt, T. Is there a resource constraint related to lithium ion batteries in cars? *The International Journal of Life Cycle Assessment* **22**, 40-53 (2017).
30. de Koning, A., Kleijn, R., Huppes, G., Sprecher, B., van Engelen, G. & Tukker, A. Metal supply constraints for a low-carbon economy? *Resources, Conservation and Recycling* **129**, 202-208 (2018).
31. Deetman, S., Pauliuk, S., van Vuuren, D. P., van der Voet, E. & Tukker, A. Scenarios for Demand Growth of Metals in Electricity Generation Technologies, Cars, and Electronic Appliances. *Environmental Science & Technology* **52**, 4950-4959 (2018).
32. Harvey, L. D. D. Resource implications of alternative strategies for achieving zero greenhouse gas emissions from light-duty vehicles by 2060. *Applied Energy* **212**, 663-679 (2018).
33. Helbig, C., Bradshaw, A. M., Wietschel, L., Thorenz, A. & Tuma, A. Supply risks associated with lithium-ion battery materials. *Journal of Cleaner Production* **172**, 274-286 (2018).
34. Vaalma, C., Buchholz, D., Weil, M. & Passerini, S. A cost and resource analysis of sodium-ion batteries. *Nature Reviews Materials* **3**, 18013 (2018).
35. Ziemann, S., Müller, D. B., Schebek, L. & Weil, M. Modeling the potential impact of lithium recycling from EV batteries on lithium demand: A dynamic MFA approach. *Resources,*

Conservation and Recycling **133**, 76-85 (2018).
