# Supplementary material for: Impact of transport electrification on critical metal sustainability with a focus on the heavy-duty segment
Source: Nat Commun. 2019 Nov 27;10:5398. doi: 10.1038/s41467-019-13400-1 (PMC6881386; doi:10.1038/s41467-019-13400-1)
Supplement: Supplementary file 3 — Reporting Summary [file 41467_2019_13400_MOESM3_ESM.pdf]

nature research

Corresponding author(s): Yong Geng

Last updated by author(s): Oct 29, 2019

Reporting Summary

Nature Research wishes to improve the reproducibility of the work that we publish. This form provides structure for consistency and transparency in reporting. For further information on Nature Research policies, see Authors & Referees and the Editorial Policy Checklist.

Statistics

For all statistical analyses, confirm that the following items are present in the figure legend, table legend, main text, or Methods section.

n/a	Confirmed	
☒	☐	The exact sample size (*n*) for each experimental group/condition, given as a discrete number and unit of measurement
☒	☐	A statement on whether measurements were taken from distinct samples or whether the same sample was measured repeatedly
☒	☐	The statistical test(s) used AND whether they are one- or two-sided<br>*Only common tests should be described solely by name; describe more complex techniques in the Methods section.*
☒	☐	A description of all covariates tested
☒	☐	A description of any assumptions or corrections, such as tests of normality and adjustment for multiple comparisons
☒	☐	A full description of the statistical parameters including central tendency (e.g. means) or other basic estimates (e.g. regression coefficient) AND variation (e.g. standard deviation) or associated estimates of uncertainty (e.g. confidence intervals)
☒	☐	For null hypothesis testing, the test statistic (e.g. *F*, *t*, *r*) with confidence intervals, effect sizes, degrees of freedom and *P* value noted<br>*Give P values as exact values whenever suitable.*
☒	☐	For Bayesian analysis, information on the choice of priors and Markov chain Monte Carlo settings
☒	☐	For hierarchical and complex designs, identification of the appropriate level for tests and full reporting of outcomes
☒	☐	Estimates of effect sizes (e.g. Cohen's *d*, Pearson's *r*), indicating how they were calculated

Our web collection on statistics for biologists contains articles on many of the points above.

Software and code

Policy information about availability of computer code

Data collection	No software was used.
Data analysis	Matlab R2015b<br>Excel 2016

For manuscripts utilizing custom algorithms or software that are central to the research but not yet described in published literature, software must be made available to editors/reviewers. We strongly encourage code deposition in a community repository (e.g. GitHub). See the Nature Research guidelines for submitting code & software for further information.

Data

Policy information about availability of data

All manuscripts must include a data availability statement. This statement should provide the following information, where applicable:

- Accession codes, unique identifiers, or web links for publicly available datasets
- A list of figures that have associated raw data
- A description of any restrictions on data availability

Data associated with the paper can be found at https://doi.org/10.6084/m9.figshare.10062512. The source data underlying Figs. 1-2, Supplementary Figs. 1-12, Supplementary Table 2 are provided as a Source Data file.

Field-specific reporting

Please select the one below that is the best fit for your research. If you are not sure, read the appropriate sections before making your selection.

☐ Life sciences ☐ Behavioural & social sciences ☒ Ecological, evolutionary & environmental sciences

For a reference copy of the document with all sections, see nature.com/documents/nr-reporting-summary-flat.pdf

Ecological, evolutionary & environmental sciences study design

All studies must disclose on these points even when the disclosure is negative.

Study description	The lithium inflow, outflow and stock associated with electric vehicles are simulated by using the Transport Impact Model (TIM) developed by the China Automotive Energy Research Center at Tsinghua University. TIM is a technology-rich, bottom-up approached model that is used to simulate the energy, environmental, and resource impacts from the global transport sector. The simulation covers 140 countries (or special regions) and five vehicle types (passenger vehicles and light-duty commercial vehicles are categorized into the LDV segment; medium-duty trucks, heavy-duty trucks and heavy-duty buses are categorized into the HDV segment), with a one-year step length from 2000-2100.
Research sample	This study covers 140 countries that have records of vehicle sales. Other countries are ignored due to data limitation and their little impact on global resource dynamics.
Sampling strategy	Our research is not based on experiments, so this report item does not apply to our research.
Data collection	This study uses a bottom-up modeling approach, and the database is established based on a wide range of existing data sources.
Timing and spatial scale	The temporal scale of the simulation is 2000-2100. The spatial scale of the simulation is 140 countries that have records of vehicle sales.
Data exclusions	No data exclusion.
Reproducibility	Our research is not based on experiments, so this report item does not apply to our research.
Randomization	Our research is not based on experiments, so this report item does not apply to our research.
Blinding	Our research is not based on experiments, so this report item does not apply to our research.

Did the study involve field work? ☐ Yes ☒ No

Reporting for specific materials, systems and methods

We require information from authors about some types of materials, experimental systems and methods used in many studies. Here, indicate whether each material, system or method listed is relevant to your study. If you are not sure if a list item applies to your research, read the appropriate section before selecting a response.

Materials & experimental systems

n/a	Involved in the study	
☒	☐	Antibodies
☒	☐	Eukaryotic cell lines
☒	☐	Palaeontology
☒	☐	Animals and other organisms
☒	☐	Human research participants
☒	☐	Clinical data

Methods

n/a	Involved in the study	
☒	☐	ChIP-seq
☒	☐	Flow cytometry
☒	☐	MRI-based neuroimaging
